# Transitional care in skilled nursing facilities: a multiple case study

**DOI:** 10.1186/s12913-016-1427-1

**Published:** 2016-05-17

**Authors:** Mark Toles, Cathleen Colón-Emeric, Mary D. Naylor, Julie Barroso, Ruth A. Anderson

**Affiliations:** University of North Carolina at Chapel Hill, School of Nursing, 7460 Carrington Hall, Chapel Hill, NC 27599 USA; School of Medicine and the Geriatric Research, Education and Clinical Center (GRECC), Durham Veterans Affairs Medical Center, Duke University, DUMC 3469, Durham, NC 27710 USA; NewCourtland Center for Transitions and Health, University of Pennsylvania School of Nursing, Room 341 Fagin Hall, 418 Curie Blvd., Philadelphia, PA 19104-4217 USA; Medical University of South CarolinaCollege of Nursing, Room 508 99 Jonathan Lucas St., Charleston, SC 29425-1600 USA; University of North Carolina at Chapel Hill, School of Nursing, 7460 Carrington Hall, Chapel Hill, NC 27599 USA

**Keywords:** Older adults, Transitional care, Skilled nursing facilities

## Abstract

**Background:**

Among hospitalized older adults who transfer to skilled nursing facilities (SNF) for short stays and subsequently transfer to home, twenty two percent require additional emergency department or hospital care within 30 days. Transitional care services, that provide continuity and coordination of care as older adults transition between settings of care, decrease complications during transitions in care, however, they have not been examined in SNFs. Thus, this study described how existing staff in SNFs delivered transitional care to identify opportunities for improvement.

**Methods:**

In this prospective, multiple case study, a case was defined as an individual SNF. Using a sampling plan to assure maximum variation among SNFs, three SNFs were purposefully selected and 54 staff, patients and family caregivers participated in data collection activities, which included observations of care (*N* = 235), interviews (*N* = 66) and review of documents (*N* = 35). Thematic analysis was used to describe similarities and differences in transitional care provided in the SNFs as well as organizational structures and the quality of care-team interactions that supported staff who delivered transitional care services.

**Results:**

Staff in Case 1 completed most key transitional care services. Staff in Cases 2 and 3, however, had incomplete and/or absent services. Staff in Case 1, but not in Cases 2 and 3, reported a clear understanding of the need for transitional care, used formal transitional care team meetings and tracking tools to plan care, and engaged in robust team interactions.

**Conclusions:**

Organizational structures in SNFs that support staff and interactions among patients, families and staff appeared to promote the ability of staff in SNFs to deliver evidence-based transitional care services. Findings suggest practical approaches to develop new care routines, tools, and staff training materials to enhance the ability of existing SNF staff to effectively deliver transitional care.

## Background

Following hospitalization for acute conditions, it is increasingly common for older adults in the U.S. to transition to a skilled nursing facility (SNF) for medical, nursing and rehabilitative care, after which they will transition again to home [1, 2]. In 2013, 1.7 million older adults, who discharged from U.S. hospitals, transferred to SNFs, where the average length of stay was 24 days and the total cost to Medicare was $ 28.8 billion [3]. In this study of transitional care in SNFs, it is important to note that the term “SNF” refers to nursing homes in which staff provide care before patient transitions to home or other settings.

Transitional SNF patients experience risk for poor health outcomes as they transfer between settings and providers of care [2, 4]. The advanced age, dependence on others for daily support and lower socioeconomic status of many SNF patients contribute to their vulnerability to poor health outcomes; for example, in 2013, 73 % of SNF patients were aged 75 years or older, 49 % had limitations in 3 to 6 activities of daily living (such as dressing, bathing, and eating), and 37 % were dually eligible for Medicare and Medicaid [5]. After transitions from SNFs to home, patients and their primary caregivers often struggle to self-manage health care tasks, such as monitoring and responding to changes in health, taking or administering the correct medications, and participating in follow-up rehabilitative and medical treatments [4]. Available data indicate that SNF patients experience poor health outcomes after transitioning from SNFs to home: 22 % required emergency services and/or hospital stays within 30 days of SNF discharge, and 39 % required emergency services and/or hospital stays within 90 days [6, 7]. This finding suggests that SNF patients and their primary caregivers (e.g. family members or friends who assist the patient) may not be prepared for care transitions from SNFs to home. It also suggests that SNF patients would benefit from transitional care services, designed to “promote coordination and continuity of care” as they transition between settings and providers of care [8, 9].

Randomized trials of formal transitional care interventions in hospitals and in one SNF have demonstrated reductions in the rate of hospital readmissions after older adults transfer to home [10–13]. These effective interventions used specialized transitional care staff to provide services before discharge (e.g., medication reconciliation and disease self-management education), services that bridged hospital and community-based care (e.g., patient-centered discharge records) and services after discharge services at home (e.g., follow-up calls or home visits) [11]. However, little is known about who delivers transitional care in SNFs, what transitional care services are provided, or how the organizational structure supports the staff members that deliver transitional care services [14, 15]. Moreover, resource limitations in SNFs likely prohibit adding transitional care staff, used in many hospital-based transitional care interventions, and data are needed to describe how existing staff members can be trained and supported to provide needed care.

Our purpose was to describe how organizational structures and staff interactions are used by existing SNF staff to deliver transitional care services. The aims of this prospective, multiple case study were to: 1) describe organizational structures in SNFs that facilitated transitional care services; 2) describe care-team interaction strategies (such as sharing information and providing feedback to solve problems) that promoted timely and complete transitional care; and 3) compare similarities and differences in transitional care services that existing staff provided for SNF patients and their primary caregivers in three SNFs. The rationale for the study was to identify targets for intervention and/or quality improvement programs to improve transitional care delivery by existing staff in SNFs, and thereby improve SNF patient outcomes after transfers to home.

### Guiding models

We drew on two existing models to guide the concepts to be described in this study. First, we used Donabedian’s Model of Health Care Quality to guide our description of (a) organizational structures (e.g., staff knowledge, tools and routines) and (b) care processes (e.g., transitional care services) [16]. See Table 1. Using the logic of Donabedian’s Model of Health Care Quality, we anticipated that organizational structures in SNFs would increase the ability of staff to deliver transitional care services [16].

**Table 1:** Conceptual model: transitional care in SNFs

Organizational Structure: Three organizational supports in SNFs that facilitate delivery of transitional care services [18].	
Structure	Definition
Staff knowledge	Professional staff members (e.g., physicians, nurses, rehabilitation therapists and social workers) are (a) available to patients and family caregivers and (b) skilled in delivering transitional care.
Care routines	Predictable schedules that staff members use to deliver transitional care, including team meetings focused on patient and caregiver needs, family and patients meetings, and cycles of care delivery and assessment to monitor outcomes.
Tools	Templates and information technology that staff use to document transitional care services and for create individualized patient and caregiver written instructions.
Care-team Interactions: Informal interactions among patients, caregivers and staff that help them form relationships (connect), exchange information, and solve problems [20, 33].	
Interaction	Definition
Connect	Staff members are (a) approachable for building relationships with patients and family caregivers; (b) pitch-in to help each other, patients and family caregivers; (c) recognize each other as care team members.
Exchange information	Staff members (a) listen to each other, patients, and family caregivers; (b) relay and verify the accuracy of new information; (c) communicate in pairs and larger groups of care-team members.
Solve problems	Staff members ask questions and give feedback to develop new information or understanding. Groups of care-team members participate in conversations to solve emerging problems in care.
Transitional Care Services: Eight evidence-based care processes that promote continuity and coordination of care as older adults transition between settings and providers of care [11–13].	
Process	Definition
Assess	Evaluates patient and caregiver preferences, strengths and needs related to health care for ensuring patients’ self-care ability and safety at home.
Plan	Creates multidisciplinary goals and measures to deliver transitional care based on assessments of patient and caregiver preferences, strengths and needs.
Engage	Collaborates with patients and caregivers to ensure that (a) implemented plans are congruent with their preferences and goals and (b) patients feel motivated to implement transition plans.
Reconcile medication	Verifies a correct medication list, using medications lists from home, hospital and SNF stays, and orders for planned care at home. Inaccuracies and errors of omission or commission are corrected.
Refer	Schedules and confirms the feasibility of services planned for care at home, e.g., MD appointments, home care, social services, rehabilitation, and tests/procedures.
Educate	Ensures that patients and caregivers have a written record and clear understanding of (a) the transition plan; (b) the name, purpose, dosage, administration, and side effects of medications, and (c) how to recognize and respond to warning signs changes in health or medical conditions.
Transfer	Sends timely and accurate summaries of SNF care and plans for the transition home to community providers of care.
Follow-up	Provides follow-up phone calls or home visits to promote patients’ and family caregivers’ implementation of transition plans at home.

Organizational structures were defined as the supports in SNFs that are available for transitional care services [17, 18]. As described in Table 1, three organizational structures were considered, including (a) staff knowledge of transitional care and staff understanding of the importance of providing transitional care, (b) care routines (such as scheduled meetings to plan transitional care services) and (c) tools (such as templates in the electronic medical record for documenting care). In the three SNFs studied, we examined how qualitative differences in staff knowledge, care routines and tools were related to the way staff delivered transitional care services.

While the organizational structures provide supports for providing transitional care, it is the quality of the interactions among members of patient care-teams that facilitate individualized care to be planned and implemented [19, 20]. For example, in a prior study, we found that transitional care was embedded in the informal interactions between providers and patients, and that staff more effectively delivered transitional care services when they worked in designated teams, rather than alone or in dyads [21]. Thus, we drew on Anderson et al.’s Model of Local Interaction [19], to guide our descriptions of staff interactions (see Table 1). Based on the Model of Local Interaction, we anticipated that care-team interactions promoting strong connections among team members, new information exchange, and team problem-solving would also promote the ability of staff to effectively deliver transitional care services. Thus we defined care-team interactions as behaviors (Table 1) that care-team members used to (a) connect with others (such as pitching in to help others), (b) exchange information (such as explaining the meaning of information), and (c) solve problems in care (such as including diverse team members in decision making activities) [19]. In the three SNFs studied, we examined how qualitative differences in care-team interactions were associated with the ability of staff to deliver transitional care services.

Together, high quality organizational structures and care-team interactions will allow staff to deliver transitional care services, which are the specific services to promote continuity and coordination of care as older adults transition between settings and providers of care [11, 13]. As described in Table 1, we defined eight transitional care services that have been associated with improved patient outcomes after transitions from hospitals to home [11, 13]. In the three SNFs studied, we examined whether these eight transitional care services were used to prepare individual patients for transitions from the SNF to home.

## Methods

We used a prospective, multiple case study design to describe the delivery of transitional care services in a purposefully selected sample of three SNFs [22, 23]. In this study, we (a) collected and analyzed data from observations, interviews and document reviews from multiple study participants at multiple points in time and (b) described qualitative differences in organizational structures, care-team interactions and transitional care services in the sample of cases. We defined a “case” as a SNF; within each SNF, data collection was focused on one patient, their primary family/friend caregiver(s) and the team of SNF staff who provided transitional care services. Baseline data about the organization of care in the SNF (e.g., staff roles, care routines and tools) were collected from staff; then, prospective, comprehensive case data about the care for an individual patient in each study SNF were collected from the patient’s admission to the SNF until discharge to home. All of the authors participated in the analysis and interpretation of data.

### Ethics and consent

The protocols in this study were approved by the Institutional Review Board at Duke University and underwent annual review (Pro00014871). Written permission to conduct the study was obtained from the administrator in each participating SNF. An informed consent procedure was used with all study participants, including use of a plain language statement of study procedures and risks. All individuals who provided study data signed an informed consent.

### Setting and participant sample

As described in Table 2, a sample of three SNFs was purposefully selected, based on variations in facility size, profit status, star-ratings in Nursing Home Compare (a federally administered database describing the quality of care in U.S. nursing homes) [24], and the percentage of Medicaid eligible patients in the facility. These three SNFs were selected, as a form of maximum variation sampling [25], because we believed their differences would allow us to address the study aims and generate meaningful hypotheses to guide further research.

**Table 2:** Description of SNFs and study participants ^a^

Case	1	2	3
SNF Characteristics			
Ownership	Private	Chain	Chain
Profit status	For profit	For profit	For profit
Size (bed count)	100 - 150	<100	>150
% Medicaid	<20 %	>50 %	>50 %
Nursing Home Compare	5 stars	4 stars	1 star
Patients Characteristics			
Patient age/gender	75 yrs./female	78 yrs./female	69 yrs./female
Patient length of stay	28 days	20 days	20 days
Patient medical condition	Cervical fusion and multiple health conditions	Kidney failure and multiple health conditions	Lumbar fusion and multiple health conditions
Primary Caregiver Characteristics			
Enrolled	None Available	Yes	Yes
Relationship to patient	n/a	Daughter	Daughter
SNF Staff Characteristics			
Staff care-team members^a^	LPN, MD, SW, NP, OT, PT, ARN	SW, RN, LPN, MD, OT, PT, RD, CC	SW, LPN, NP, RN, MD, OT, PT
Managers and Department Heads	8 enrolled	9 enrolled	8 enrolled

^a^ *SW* social worker, *LPN* licensed practical nurse, *RN* registered nurse, *MD* medical doctor, *NP* nurse practitioner, *OT* occupational therapist or assistant, *PT* physical therapist or assistant, *RD* registered dietician, *CC* care coordinator, *ARN* administrative nurse

In each SNF, one patient was recruited. The inclusion criterion for patients was the ability to speak in English (as indicated by the medical record and conversations during recruitment activities). Patents were excluded if they had more than mild cognitive impairment (as indicated by the description of the patient’s mental status in the hospital and SNF medical records, a verbal report provided by the director of nursing indicating the patient’s understanding of treatments and plans, and the patient’s ability to fully participate in study recruitment and consenting procedures). The first patient admitted to the SNF who met the inclusion criterion was recruited by the lead author. No patients declined to participate. A total of three patients, one from each SNF, participated in the study (see Table 2). Primary caregivers (e.g., family members or friends) were recruited if they spoke English, as indicated during recruitment conversations. Two primary caregivers participated in the study and none declined to participate (see Table 2). Staff members were recruited if they were willing to participate in the study; these included (a) department managers (such as director or assistant director of nursing, department heads of rehabilitation or social services, or medical directors) or staff who directly provided elements of transitional care with the recruited SNF patient (such as nurses, social workers and rehabilitation therapists). A total of 49 staff participated: 15 in SNF 1, 17 in SNF 2, and 17 in SNF 3 (see Table 2). Two staff members declined to participate in the study.

### Data collection

Using procedures and instruments from prior research [21], data were collected over approximately 10 weeks in each SNF in three phases (see Table 3 for an illustration of the data collection schedule in SNFs). In weeks 1-3, data were collected by observing scheduled meetings, reviewing policy and procedure documents, interviewing department heads and selected clinical staff for the purpose of describing the plan or supports for providing transitional care in each SNF (see Table 3). In weeks 4-8, the SNF patient, primary caregiver and direct care staff were recruited and data were collected (e.g., interviews, observations and document or chart reviews) to describe how staff delivered transitional care over time with an individual patient. In weeks 9-10, final data collection activities were completed, including chart and document reviews and exit interviews with department heads. The lead author collected data six days per week in each SNF for the duration of the patient’s SNF stay.

**Table 3:** Illustration of the data collection schedule in SNFs^a^

Subject or Data Source	Week																					
	1-3	4	5-7																			8-9
			1	2	3	4	5	6	7	8	9	10	11	12	13	14	15	16	17	18	19	
Interviews and Observations																						
Patient		r	c	f			o	o		o		f		o		o		o	o	f		
Caregiver		r	c			f		o							o					f		
OT		r	c	f	o					o				o			o		o	f		
PT		r	c		f		o						o				o		o	f		
SW	c, f	r	c		f	o				o									o		f	
LPN		r	c					o			o				o			o			f	
NP	c, f	r	c		o							o					o				f	
MD		r	c	o									o								f	
RN		r	c			f	o					o	f			o						
Other	c, f		c			o												o				f
Other data sources																						
Meetings	o		o	o	o	o	o			o	o	o	o	o			o	o	o	o	o	
Chart		d	d	d	d	d	d	d		d	d	d	d	d	d		d	d	d	d	d	d
Document	d		d		d		d					d		d	d			d			d	d

Key: *r* recruitment, *c* consent, *f* formal interview, *o* observation and informal interview, *d* chart or document review, *OT* occupational therapist, *PT* physical therapist, *SW* social worker, *LPN* licensed practical nurse, *NP* nurse practitioner, *MD* medical doctor, *RN* registered nurse, Other = managers, department heads, administrators

^a^Illustrates the data collection schedule for a case in which the patient length of stay was 19 days. In weeks 1- 3, we assessed organizational structure. In week 4 we recruited the patient and staff. In weeks 5-7 we did field work for 19 days with the individual patient, primary caregiver and staff. In weeks 8-9, we concluded data collection activities

### Semi-structured interviews

Semi-structured interviews were conducted in private to assure confidentiality and minimal disturbances. They were approximately 30 min in length, audio recorded, and transcribed. Transcripts were compared to audio recordings of the interviews and assessed for accuracy. In weeks 1-3 and 9-10 in each SNF, interviews were used to explore the department managers’ perceptions of the organizational supports for delivering transitional care services. Interview questions were developed to examine the main concepts and relationships in the guiding models for the research. The topic list for these interviews included procedures for assessing patient needs, organizational supports for coordinating care and team work among direct care providers. Semi-structured interviews were also used to explore perceptions of staff members who delivered transitional care services. The topic list for staff interviews included strategies for engaging patients and primary caregivers, understanding of evidence-based transitional care services, use of organizational supports to deliver care, and care-team interactions. Semi-structured interviews with patients and primary caregivers were used to describe their experiences in the SNF. The topic list for patient and primary caregiver interviews included expected transitional care outcomes, participation in transitional care services, and preparedness for discharge from the SNF to home.

### Observations and document reviews

The lead investigator directly observed staff members, patients and primary caregivers as transitional care services were delivered in the SNFs (see Table 3) [21]. Observations included “chance encounters” (e.g., interactions among staff or patients and staff in the hall or at the nursing stations) [19], team meetings such as care plan meetings, scheduled appointments between staff, patients and /or family caregivers, and one-to-one encounters, such as therapy sessions and nursing rounds. The lead author’s recorded observations in field notes which were transcribed daily in formal field notes [23, 26].

Document reviews of procedure manuals, medical charts and/or discharge documents were completed daily. Tools for reviewing documents were developed to assess the main concepts in the guiding models for the research. The purpose of document reviews was to record (a) written communication about transition needs, goals, implementation of goals, and instructions to guide care at home and (b) policies and procedures for providing transitional care services.

### Data analysis

Data were organized for analysis in Atlas.ti (Scientific Software Development, Berlin, Germany) [27]. We used thematic analysis, a method of qualitative analysis in which data are labeled with codes and categorized to identify broader ideas or “themes” within the entire data set [22, 28]. Interview transcripts, field notes and case documents were reviewed and then coded using the concepts and definitions identified in Table 1 (e.g., organizational supports, care-team interactions, transitional care services) [22, 28]. A second analyst coded a random sample of 10 % of the data to evaluate the reliability of the coding; agreement between data coders was 96 % [28]. To address Aim 1, data coded as organizational structures and transitional care services were cross-tabulated in data matrices; similarities and differences in organizational supports and transitional care services were described within and across SNFs [22]. For example, data coded as staff knowledge (an organizational structure) were cross-tabulated with data coded with each of the eight transitional care activities; then summaries of the data describing staff knowledge and each transitional care activity were written within and across SNFs. Similarly, for Aim 2, data coded as care-team interactions and transitional care services were cross-tabulated in matrices; then similarities and differences in care-team interactions and transitional care services were described within and across SNFs [22]. To address Aim 3, data coded as transitional care services were summarized and used to write narratives describing transitional care services that existing staff provided within and across SNFs. After data were analyzed, study findings were presented to SNF staff members in SNF 1 and 2; even with repeated attempts to schedule, staff members in SNF 3 were not available. In SNF 1 and 2, staff concurred with main findings and used the findings to discuss opportunities to engage in performance improvement. Finally, narratives were written to describe the main findings for each study aim. All investigators participated in thematic analysis.

## Results

The sample of participants in three SNFs included three patients, two primary caregivers and 49 staff members (see Table 2). The data from the case studies included 66 interview transcripts, 235 field notes, and 35 case documents. A total of 527 sections of text from the interviews, field notes and case documents were coded as organizational structures, care-team interactions or transitional care services. Analysis of the coded data revealed three main themes: (a) organizational support and staff members who delivered transitional care services, (b) care-team interactions and delivery of transitional care services, and (c) the challenge in providing evidence-based transitional care services. Main findings are described sequentially by theme below.

### Theme 1: Organizational support and staff members who delivered transitional care services

We found that staff more effectively delivered transitional care services when organizational structure was available to inform or coordinate their work as a team. A physician in SNF 1 noted the benefits of strong organizational support.

“I have worked in facilities where the patient is ready to go and nobody knows it. But with all our meetings and hand-offs here, you know everything that you need for all the prescriptions, all the equipment, what I need to teach the patient. So it is usually very, very planned.” (Physician)

Below, we provide specific descriptions of staff knowledge, care routines and tools that appeared to have the greatest impact on the ability of staff to deliver transitional care services.

1. Staff Knowledge

In SNF 1, a physician, social workers, rehabilitation therapists, and selected nurses understood the need for transitional care and were skilled in preparing the patient to transfer home. For example, the social worker recognized the need to help families understand routines in the SNF and the expected discharge date.

“My job is to…negotiate between the rehabilitation time under Medicare, what the patient is looking for, what the family is looking for, and to figure-out how to get everybody on the same page.”

In SNF 1, we also observed that staff members skillfully taught the patient to how to self-manage her care at home. For example, near the day of discharge, the patient clearly articulated changes in her medications and what to expect at home.

“They have been watching my Dilantin levels here and I am told they have adjusted that medicine and that my levels will return to normal. My doctor knows about that too.” (Patient)

“I have been using OxyContin for pain but that is changing too. I am starting Tylenol now and I will take that when I get home as well.” (Patient)

In SNFs 2 and 3, however, staff members had little understanding of evidence-based transitional care services. For example, a nurse misinterpreted the definition and purpose of transitional care.

“It’s a way to care for those people that are going to stay with us…we would transition them from one of our units to another.” (Nurse)

In these facilities, gaps in staff knowledge were associated with omissions in transitional care services. Most commonly, staff did not effectively engage and train family caregivers. In SNF 2 and 3, on the day of discharge, caregivers described unmet needs for information about caring for their family members at home.

“I wish I knew more about what to look for if [patient] started getting signs of getting sick or her health started failing again; what to do and who to call on for that.” (Family Caregiver)

“I know they worked with my mother but they should bring a family member in too and teach them what we should be able to do…to help this person to maneuver around like they did in the facility.” (Family Caregiver)

2. Care Routines and Tools

In SNF 1, staff members used a schedule of routine meetings to plan and implement transitional care services. These included intra-departmental meetings (e.g., rehabilitation, social work and nursing) to identify the patient’s needs for care at home, inter-departmental meetings to develop comprehensive assessments, and “family meetings” with patients, primary caregivers and SNF staff.

“We learned that it really does help to have the family meeting early in the admission because, if we don’t, there is not enough time to get the assessment, plan, or know what is going to be needed at home.” (Director of Nursing)

In SNF 1, we also found that staff used specific tools in the medical record to document and schedule events in care. For example, in our review of the patient’s medical record, we saw that social workers and rehabilitation therapists used standardized templates to document patient needs, the plan for care at home, and the patient’s ability to walk and provide self-care.

In SNFs 2 and 3, however, staff members worked without care routines and tools which limited their ability to coordinate delivery transitional care services. In SNF 3, a care plan meeting was never convened. In SNF 2, a care plan meeting with patient, primary caregiver and SNF staff was convened, but two days before the patient returned home. In this SNF, the director of nursing described the need to plan transitions in care earlier.

“We need more timely planning…we can say on Monday that this patient is discharging next Monday…then Thursday comes and the rush kicks in. It doesn’t work.” (Director of Nursing)

In SNFs that lacked care routines for delivering transitional care services, last minute plans and instructions were confusing to patients and primary caregivers. In SNF 3, at the time of discharge to home, the patient was angry about confusion in her medication orders.

“I don’t know what they are saying here. I will go see my own doctor when I get home. She will tell me what I need to take.” (Patient)

Finally, across the SNFs, we found gaps in care routines and tools; for example, we did not observe staff that coordinated transitional care using templates to guide medication reconciliation, standardized care routines for transferring information to primary care, or electronic medical record systems to integrate work among staff in different disciplines.

### Theme two: care-team interactions and delivery of transitional care services

Across the SNFs, we found that the informal interactions among individual patients, primary caregivers and staff allowed individualized care to be planned and implemented. Below, we describe interactions which appeared to have the greatest impact on the ability of staff to deliver transitional care services.

1. Interactions associated with Connections and Information Exchange on Care-teams

In SNF 1 and inconsistently in SNF 2, staff members were approachable to others and pitched in to help each other implement transitional care services. In SNF 1, staff members described the importance of being approachable and sharing information.

“I like to be available…I want [staff] to know they can come to me and ask for help…” (Nurse)

“We have our rehabilitation meeting on Tuesday and everybody gets together and assesses what the patient needs when they are discharged…I would say that is our greatest strength here, having that open communication between all the disciplines.” (Quality Assurance Nurse)

In SNF 1, we found that the easy flow of information contributed to effective transitional care services, for example, a nuanced and patient-centered assessment of the patient’s preparedness for returning home. A social worker described her understanding of the patient’s preferences, and how the team was working to honor them.

“Some folks need to feel like they are driving the bus – for [patient name], we need to let her do that. The physical therapist…realized that the cervical collar was really the big issue – because she can’t see her feet when she wears it. We are very concerned to reduce her risk of falls, so we need to follow the therapist’s advice; we will work with her here until the collar is off.” (Social Worker)

In SNFs 2 and 3, however, where care-team interactions were observed less often, staff delivered transitional care in isolation of other team members and habitually worked alone, or in pairs with patients.

“It’s just easier for me to do this [discharge planning] myself than to try to coordinate this work with someone else.” (Nurse Care Coordinator)

Two days later, the same nurse described a backlog of transitional care services and was overwhelmed by her workload.

“I’m running around like a chicken with its head cut-off. The [Agency Name] did not order Oxygen – now on top of everything else, I have to figure that out.” (Nurse Care Coordinator)

In SNF 2, a physical therapist lamented barriers to communication that limited her ability to follow-up on patient and caregiver needs.

“We write notes, but I do not think they are closely followed [by other staff]. So what I have to do is try to find the family…it is hard because they are not here when I am here…I can tell the rehabilitation director [what to tell them], but that is not a guarantee that it will make it to the family.” (Physical Therapist)

In these SNFs, staff members were sometimes afraid to provide feedback and circumvented conversations with key members of the care-team. For example, one department head related how staff avoided the physician and expected her to communicate with him for the group.

“If [staff members] have any issues and think [physician name] can help, they come to *me*, and say ‘can you, you know, talk to him about it.’” (Director of Nursing)

In SNF 2 and 3, limitations in care-team interactions were associated with less effective information flow among team members, which limited the quality of transitional care services. In SNF 2 for example, a care coordinator planned a discharge date with the patient; yet, two days before the discharge, the occupational therapist and physician were not aware the discharge was pending.

2. Interactions associated with Problem Solving on Care-teams

In SNF 1, care-team members proactively approached problems and conflicts in overlapping subgroups, including the patient, occupational therapist and nurse; the physician, social worker and nurse; and others. A nurse explained how team interactions helped her to prepare the patient for returning home.

“Just having [team members] there gives us a chance to solve individual problems and to hear what the patient’s or family member’s questions are. This gives a chance to talk about the plan and prevent some of the last minute questions…” (Nurse)

Problem-solving interactions on the care-team appeared to support the coordination of transitional care services. For example, the social worker assessed the need to help the patient change agencies for home health aides; she solved problems with the patient and community social worker; and she relayed feedback to and from other care-team members in the SNF. The patient appreciated well-coordinated transitional care.

“They helped set things up at home…even with the holidays, they found out what was needed, like learning my community worker’s name and working with him…I was worried. I would not have been able to handle it all.” (Patient)

However, limited connections and information exchange on care-teams in SNFs 2 and 3 appeared to constrain the degree that team members worked together to solve problems; rather, one or two clinical experts appeared to solve problems for the entire team. In SNF 2, for example, the director of nursing – who supervised all clinical staff in the SNF - directly intervened to address an impasse in discharge planning. The director of nursing convened a family meeting and led a conversation about negotiating care with the patient at home.

“You have to teach [patient name] to compromise…We are asking her to make some big changes…You know, work with her so she can learn.” (Director of Nursing)

Though ultimately effective, the director of nursing’s intervention was an act of crisis management, two days before discharge, when she learned that staff had neither met with the caregivers nor discussed the conflicts in the family that limited transition planning.

Across the study SNFs, we observed significant gaps in problem solving. Most importantly, conversations frequently did not include interactions with family caregivers, even though they were present in the facilities and expressed a desire for greater participation.

### Theme 3: The challenge in providing evidence-based transitional care services

We found wide variation in transitional care services, but only one of three SNFs was able to deliver most of the recommended services using existing staff. Of the potential transitional care services described in the literature, we found that staff in all 3 SNFs: (a) reconciled medications lists from the hospital and the SNF, (b) taught patients strategies for safe physical function, (c) provided verbal instructions for medication administration, (d) referred patients for home care, and (e) recommended follow-up with primary care providers. Across the study SNFs, staff *inconsistently*: (a) assessed patient goals and needs at home, (b) reconciled medication lists from the SNF and home, (c) assessed primary caregiver needs or engaged primary caregivers in treatment plans, (d) taught written transition plans and (e) provided detailed written instructions for care at home. Finally, no SNF staff members transferred treatment information to follow-up medical providers or visited/called patients or caregivers at home to reinforce transition plans.

In SNF 1, compared to SNFs 2 and 3, we found that staff delivered substantially more transitional care services, and more effectively prepared the patient for continuing care at home (see Table 4). In the narrative that follows we describe how staff in SNF 1 delivered transitional care services, to highlight practices that might inform future interventions to improve transitional care in SNFs.

**Table 4:** Findings: transitional care services provided by week of patient admission

SNF	Week 1	Week 2	Week 3	Week 4
1	• Thoroughly assessed needs for care at home • Reconciled medication lists • Taught medications and treatment goal	• Team meeting to engage patient and plan care at home • Contacted community physicians to plan medical goals	• Team taught self-management skills; • Social worker referred and activated community supports • MD taught goals and medications	• Provided written instructions • Scheduled MD follow-up • Did not transfer records to follow-up MD or contact patient at home
2	• Assessed functional needs • Did not assess patient/caregiver gaps in knowledge about dialysis or medications • Reconciled only hospital and SNF medication lists	• Rehabilitation therapists taught the patient (but not caregivers) • Did not engage family members in planning for the patient’s complex needs at home	• Assessed gaps in discharge and self-care ability for the patient at home • Team meeting to plan care at home • MD reconciled medications and taught the patient	• Referred support for new dialysis • Partial written instructions provided • Did not schedule MD follow-up, transfer records to follow-up MD or contact patient at home
3	• Assessed risk for falls and need for lower extremity rehabilitation. • Did not reconcile medication lists, identify caregiver needs, or address recent cardiac changes	• Planned care in SNF but did not plan the transition to home • Did not engage family members • The patient disengaged from SNF staff and planned goals with primary care	• The patient scheduled MD follow-up • Did not create integrated plan for care at home • Did not contact the community social worker • Taught self-management for mobility and transfer safety	• Referred home care • Taught a written list of discharge medications • Did not schedule MD follow-up, transfer records to primary care or contact the patient at home

First, early in the SNF stay, staff assessed the patient’s transition needs (such as changes in medications and environmental safety at home) and learned the patient’s preferences, for example, the patient’s hope to remain in the SNF until her neck brace was discontinued. Second, staff consistently taught the patient strategies for safely walking, washing, and cooking at home; for example, an occupational therapist used role-play kitchen activities to teach the patient, who subsequently noted how to use her walker at home.

“I understand now. I can hold on [to] this thing even when I reach something up here.” (Patient)

Third, staff consistently re-assessed the patient’s response to treatment, which fostered their ability to plan the transition home. For example, midway through the patient’s stay, a social worker recognized the patient’s increasing confidence.

“I feel like she [the patient] is coming into her own…She had the care plan meeting…then, physical therapy pulled her in [and taught her safety skills]…and she understands now what her goal is…I think she feels a whole lot better [about going home].” (Social Worker)

Fourth, staff wrote detailed instructions and used them to reinforce teaching about care at home, including medication administration, scheduled appointment names and dates, contacts for community support and home care services, and a list of warning signs that indicated the need to follow-up with a medical provider. On the day of discharge, the patient was very satisfied with transitional care in the SNF.

“This is a very, very, very overall equipped convalescent facility… everyone here pays attention to what you need, every detail.” (Patient)

## Discussion

In a sample of three purposefully selected SNFs, we found that existing staff members in one SNF effectively delivered evidence-based transitional care services, while those in two facilities provided more fragmentary transitional care that inconsistently addressed the goals of patients and their primary caregivers. An earlier study indicated that 22 % of older adults either visited an emergency department without hospitalization or were re-hospitalized within 30 days of discharge from a SNF [6]. The current study suggests an explanation for these findings by describing gaps and inconsistencies in transitional care services that may contribute to poor outcomes for older adults in SNFs after they return home. Findings in the study do not account for differences in income, community support, primary care, and other factors that might contribute to poor outcomes. However, our findings of gaps in transitional care services suggest that poor outcomes may be related to modifiable strategies for delivering care in SNFs. This finding also suggests the need to test the feasibility and effectiveness of evidence-based transitional care for SNF patients.

Our finding, that one SNF with robust organizational structure and care-team interactions more effectively delivered transitional care services than two SNFs with limited organizational structure and care-team interactions, suggests two strategies for improving the way existing staff deliver transitional care. The first strategy is to develop organizational structure in SNFs that supports staff members who deliver transitional care; for example, new procedures in SNFs for creating written self-care plans for patients to use at home. The second strategy is to develop interactions on patient care-teams that promote connections, information exchange and problem solving; for example, carefully listening to family caregivers to evaluate their understanding of new instructions for care at home. In two prior studies, transitional care services were associated with reductions in 30 day hospital readmissions of SNF patients [10, 18]. Our findings potentially extend these studies and suggest that strengthened supports and training for staff who deliver transitional care are needed to reduce hospital readmissions after SNF patients return home.

These findings suggest practical implications for policymakers and research. In 2014, the U.S. Congress passed a value based purchasing program for SNFs, which will create penalties for SNFs (based on Medicare reimbursement rates for care in SNFs) to prevent avoidable re-hospitalizations of SNF patients 30 days after discharge [3]. A first step to improving transitional care, and potentially patient outcomes, might be to develop staff knowledge about care transitions, including training to help staff: (a) recognize that SNF patients are at risk for poor outcomes during transitions to home and (b) learn the importance of key transitional care steps for minimizing these risks [29, 30]. The frequently observed focus on “discharge planning” in the last 2-3 days of SNF stays suggests that staff do not appreciate the scope and importance of transitional care. With greater awareness, existing staff in SNFs may be receptive to the changes in workflow and additional steps needed to implement evidence-based transitional care interventions and prevent avoidable re-hospitalizations after patients return home [31, 32]. Training materials in the Re-Engineered Discharge (RED) Toolkit are a useful starting point to develop new educational materials [9, 10, 32]; in particular, Tools 3 - 6 which describe transitional care services associated with reduced rates of hospital readmissions, such medication reconciliation, education strategies for assuring that patients understand new healthcare instructions, written transition plans for use at home, and calls or visits to patient homes after discharge [13, 32].

The findings also suggest the need for new tools and procedures to plan patient transitions from the SNFs to home [9, 18]. We found that staff in two SNFs delivered many transitional care services in the last 24 to 72 h of patient stays; this suggests the need to start transitional care services earlier and establish schedules to move staff stepwise through a process to prepare SNF patients and caregivers for discharge. Moreover, data are not currently available which describe ways that electronic medical records might be adapted in SNFs to support timely and collaborative work of physicians, social workers, nurses and rehabilitation therapists. As financial pressures continue to reduce the average length of stay in SNFs, organizational supports, such as new tools in electronic medical records systems, will be important resources for improving the timeliness and number of team member contributions to transitional care services. New transitional care tools in electronic medical records systems may also be useful to managers and department heads in SNFs who oversee performance improvement and quality assurance in SNFs. The transitional care practice guidelines developed by the American Medical Directors Association includes tools that SNF managers might use to monitor the quality of transitional care services; for example, the frequency of caregiver inclusion in care plan meetings, hand-offs of clinical information to follow-up physicians, and post-discharge follow-up calls or visits [29].

Finally, our findings have implications for practice and research about patient- and family- centered care-teams in SNFs. Consistent with earlier studies [19, 20, 21, 33], our findings suggest the need to cultivate formal and informal care-team interactions among physicians, nurses, rehabilitation therapists and social workers who directly prepare patients for transitions in care. Findings in this study extend earlier research by identifying strategies to engage family caregivers and establish their important roles on patient care-teams. For example, the findings suggest the need to ask questions about caregiver roles and responsibilities at home; to explain new information carefully, especially critical details in planning such as the expected day of discharge; and to make time for solving problems in groups with patients and their primary caregivers. Training tools in the CONNECT for Better Falls Prevention in Nursing Homes study may help staff members recognize how their interactions with patients and primary caregivers influence the effectiveness of problem solving on patient care-teams [34]; in particular, role playing exercises in the CONNECT training tools are potentially helpful models, as they create opportunities for staff to practice giving and receiving feedback with each other and in simulated encounters with patients or primary caregivers.

These findings come with several caveats. First, the limited number of SNFs studied may limit the transferability of the findings [35, 36]. For example, differences in ownership in study SNFs may have contributed to the variations in transitional care services, organizational support and care-team interactions; quantitative studies with larger samples of SNFs are warranted to examine the relationship between facility-level factors (e.g., ownership, profit status, staffing, and patient populations) and transitional care provided in SNFs. Second, our study was designed to closely examine how differences in organizational support and care-team interactions were related to the quality of transitional care in three SNFs; it was not designed to study how differences in the needs of patients and primary caregivers – within the same SNF – may have influenced the quality of transitional care. Additional research may be needed to evaluate the relationship between SNF patient factors, such as health literacy and perceived social support, and the outcomes of transitional care services. Third, SNF staff members were aware that the study focused on transitional care, which may have influenced their behavior and introduced bias into the data. However, this limitation was attenuated by the design for data collection and analysis, which included triangulation of data in the analysis that were collected from multiple sources (e.g., observations and interviews), providers and points of time. Fourth, data were collected by the lead investigator who may have had preconceptions that biased the data collection and analysis; this limitation was managed by procedures for insuring rigor [21], in which a second coder verified 10 % of the data. Finally, the study did not examine patient outcomes at home. Despite these caveats, the study permitted prolonged and in-depth data collection in three diverse SNFs; it also facilitated a description of practical strategies to train and support existing staff in SNFs that deliver transitional care.

## Conclusions

Existing staff members can effectively deliver evidence-based transitional care services with appropriate organizational structure and care team interactions; research is needed to determine the influence of transitional care interventions on patient outcomes after SNF discharge.

### Ethics

The protocols in this study were approved by the Institutional Review Board at Duke University and underwent annual review (Pro00014871).

### Consent to participate

A written, informed consent procedure was used with all study participants, including use of a plain language statement of study procedures and risks. Individuals who provided study data signed an informed consent.

### Consent to publish

Not applicable.

### Availability of data and materials

Data supporting study findings are contained in the manuscript. Some data will not be made available as publication of the documents, field notes and interview transcripts from the case studies would compromise the confidentiality of study participants.

## Abbreviation

SNF: skilled nursing facility
